# The relationship between care burden and quality of life in caregivers of hemodialysis patients

**DOI:** 10.1186/s12882-018-1120-1

**Published:** 2018-11-12

**Authors:** Haleh Jafari, Azita Ebrahimi, Abbas Aghaei, Alireza Khatony

**Affiliations:** 1 School of Nursing and Midwifery, Kermanshah University of Medical Sciences, Kermanshah, Iran; 2 Clinical Research Development Center, Imam Reza Hospital, Kermanshah University of Medical Sciences, Kermanshah, Iran; 3 Social Determinants of Health Research Center, Research Institute for Health Development, Kurdistan University of Medical Sciences, Sanandaj, Iran; 4 Social Development and Health Promotion Research Center, Kermanshah University of Medical Sciences, Kermanshah, Iran; 5 Nursing Department, School of Nursing and Midwifery, Doulat-Abad, Kermanshah, Iran

**Keywords:** Family caregiver, Quality of life, Burden, Hemodialysis

## Abstract

**Background:**

Caregivers of hemodialysis patients endure a significant caring pressure as a result of caring for patients with chronic illness, which can affect their quality of life. Disruptions in the quality of life of these caregivers impose double pressure on them and disrupt the care process. Therefore, the present study aimed to determine the level of care burden and its relationship with quality of life of caregivers of hemodialysis patients.

**Methods:**

In this descriptive-analytical study, 246 caregivers of hemodialysis patients were enrolled by census method, so that, all patients and caregivers who attended the study environment at morning, evening and night participated in the study. The study tool was a three-part questionnaire, which included personal information, Novak & Guest Care burden Questionnaire, and WHOQOL-BREF Quality of Life Questionnaire. Data were analyzed by descriptive, statistical and inferential tests.

**Results:**

In total, 37.4% of caregivers were experiencing high and very high levels of care burden and 42.7% of them were experiencing a moderate level of care burden. The mean and standard deviation of the quality of life of caregivers was 76.27 ± 13.67 out of 130. There was a significant and negative correlation between the total scores of care burden and quality of life (*r* = − 0. 436, *P* < 0.001). The factors influencing care burden included variables such as; level of patient’s caring capability, the patient’s incidence of other chronic diseases, and the age of the caregiver. So that, in case of reduced patient’s capability in self-care, the patient’s incidence of other chronic diseases, and the increased age of the caregiver, the level of care burden on the caregivers would be increased.

**Conclusions:**

The caregivers of hemodialysis patients endure high level of care burden and this pressure has a negative effect on their quality of life. Therefore, it is recommended to pay more attention to the needs of caregivers and provide adequate social, economic, physical and psychological support for them.

## Background

The chronic kidney diseases are among the main health problems all around the world [1]. The chronic kidney failure is one of the renal disorders, which is associated with irreversible loss of kidney function [2]. There are many treatment methods for chronic kidney failure, among which, the hemodialysis is the most common method [3]. In the united stated, from every 678,000 patients who suffer from end stage renal failure, 71% use some type of dialysis [4]. In Iran, the growth rate of this disorder is about 12% per year, which is higher than the world’s average [5]. While hemodialysis prevents the death of patients with chronic renal failure [6], it causes significant changes in their lifestyle. Hemodialysis reduces the patient’s energy level and, with the frequent need for dialysis, affects their ability to work and perform everyday activities, which disrupts the normal lives of patients and their caregivers [1]. Caregivers are people who, during the course of illness and treatment, are the most involved people in the care of patients and help them to adapt and manage their chronic disease [3]. Caregivers are usually family members or friends of the patient who take care of the patients on a daily basis and support them physically, mentally and socially, but do not receive any reimbursement for the care they provide [7].

The chronic nature of kidney failure, various complications of hemodialysis treatment, and significant changes in the lifestyle of patients, cause the caregivers and family members of the patient to experience a high level of care burden, in a way that, their mental health will be influenced to various degrees [3]. In this regard, results of various studies indicate that caregivers of hemodialysis patients are under pressure physically, emotionally and financially [7, 8], and are therefore exposed to a variety of physical and psychological risks [9]. Evidence suggests that caregivers of hemodialysis patients are under high levels of care burden [3, 10, 11]. Care burden is a term used for caregivers and is a kind of distress that caregivers suffer from as a result of caring for patient [12]. It also has physical, psychological, social, and financial aspects [13]. The results of a study show that caregivers of hemodialysis patients experience significant level of care burden that affects their quality of life [14]. Increasing care burden and decreasing quality of life can lead to complications such as depression. There is also a significant relationship between increased care burden and reduced care provided by caregivers, because care burden can have a very devastating impact on individuals. The amount of stress that caregivers of a chronic patient experiences is a serious illness [11]. Although caregivers of hemodialysis patients are at risk of developing various diseases and are also known as hidden patients, they are unfortunately often ignored [12].

Care burden affects caregivers’ quality of life and may result in reduced care provision and deteriorating condition for patients with chronic illness. The deterioration of patient’s condition can increase care burden and cause a vicious cycle, and if timely intervention is not done, it may lead to a gradual exhaustion of the caregivers [1]. Therefore, timely identification of these pressures in caregivers plays a decisive role in promoting their physical and mental health [3]. Thus, conducting a study on the care burden of the caregivers of hemodialysis patients and providing appropriate strategies in this regard seems necessary. Considering the lack of knowledge on the level of care burden in the caregivers of hemodialysis patients in Kermanshah, and since reviewing this problem is the first step towards providing a solution for this issue, the present study was conducted to determine the level of care burden and its relationship with the quality of life of caregivers of hemodialysis patients.

## Methods

### Study questions

‘We sought to answer the following questions: 1) what is the level of care burden in the caregivers of hemodialysis patients?, 2) How is then status of quality of life in caregivers of hemodialysis patients?, 3) what is the relationship between care burden and quality of life in caregivers of hemodialysis patients?, and 4) what is the relationship between care burden and individual variables?

### Study design

The present study is a cross-sectional and descriptive-analytical study that was conducted in 2017 over 5 month period from May to October.

### Study samples

The research population consisted of all caregivers of hemodialysis patients attending the hemodialysis centers in Imam Reza and Imam Khomeini hospitals of Kermanshah-West of Iran. These hospitals are affiliated with Kermanshah University of Medical Sciences (KUMS). The samples included 246 caregivers of hemodialysis patients who were enrolled in the study by census method. Therefore, all patients and caregivers who attended the study environment at morning, evening and night participated in the study. Inclusion criteria were; agreeing to participation in the study, absence of diagnosed mental illness, and having history of caring for a hemodialysis patient for at least 3 months.

### Instrument

The research tools were three questionnaires including a) sociodemographics and medical conditions, b) Caregiver Burden Inventory, and c) Quality of Life questionnaire. Sociodemographic and medical conditions questionnaire, was devoted to the demographic information of patients and their caregivers, including questions about the age of the patient and the caregiver, the sex of the patient and the caregiver, the amount of income, education level, occupation, caregiver’s marital status, patient’s other chronic illness, how long have the patient been doing hemodialysis, and patient’s ability to do his/her personal tasks. In order to assess the patient’s overall ability to perform daily tasks such as bathing and doing homework, the caregiver was asked a question, and he/she had to choose one of the options of low, medium or high.

The 1989 care burden questionnaire was developed by ÂNovak & Guest to measure objective and subjective care burden. It includes 24 questions and consists of five subscales of time-dependence, developmental, physical, social, and emotional care burdens. This questionnaire was translated into Persian by Abbasi et al. and its validity and reliability have been confirmed by content validity method and Cronbach’s alpha (alpha = 0.90) respectively [3]. It has also been used in numerous studies [15–18]. Responses were in the five-option Likert’ scale, so that, the samples in response to each question, had to pick one of the five given options, which included completely incorrect (score 1), incorrect (score 2), somewhat correct (score 3), correct (score 4) and completely correct (score 5). The scores of the questionnaire ranged from 24 to 120, with scores of 24 to 47 indicating low Burden, 48 to 71 moderate Burden, 72 to 95 high Burden and 96 to 120 very high Burden [19].

The WHOQOL-BREF questionnaire was used to assess the quality of life of caregivers. Validity and reliability of this questionnaire have been assessed and verified by Nedjat et al. Also, its Cronbach’s alpha in the healthy population and non-healthy population has been calculated as 0.73, and 0.77 respectively [20]. This questionnaire has 26 questions covering four areas of Physical Health (7 questions), Psychological Health (6 questions), Social Relationship Health (3 questions) and Environmental Health (8 questions), and the first two questions are not specific to any area. The questionnaire is based on the 5-point Likert’s scale, including very bad [1] to very good [5], very dissatisfied [1] to very satisfied [5], not at all [1] to very possible [5], and never [1] to always [5]. The three-point scoring is also reversed, which in total, the range of scores is between 26 and 130. A higher score indicates a better quality of life.

### Data collection

To conduct the study, the necessary permission was obtained from the KUMS Ethics Committee. Then, the researcher attended the hemodialysis centers during all shifts and proceeded with the sampling. For this purpose, the goals of the study were first explained to the caregivers of hemodialysis patients and they agreed to participate in the study. Then, they were given the questionnaire and after completing them, the questionnaires were collected.

### Data analysis

The collected data were analyzed by 20th version of the Statistical Package for Social Sciences (SPSS v.16.0; SPSS Inc., Chicago, IL, USA) using descriptive statistics (mean and frequency percentage), and analytical statistics (Kolmogorov-Smirnov [KS], Spearman and Pearson correlation coefficients). The results of KS test showed that, the total scores of care burden and quality of life had normal distribution, but their domains did not follow this rule. Therefore, parametric statistical tests were used for this purpose. Pearson correlation coefficient was used to examine the relationship between the scores of total quality of life and total care burden. Also, Spearman correlation coefficient was used to examine the correlation between the dimensions of these two variables. In order to investigate the relationship between the background variables and the quantitative demographic variables, the simple liner regression was used and to find the same relationship with the qualitative demographic variables, the variance analysis was used. The significance level of 0.05 was considered.

### Ethics

The university’s Ethics Committee approved the study. The research conforms to the provisions of the Declaration of Helsinki in 1995 (as revised in Edinburgh 2000). The purpose of study was explained to all samples and they were assured about the confidentiality of their information. Written consent was obtained from all participants.

## Results

The mean and standard deviation of patients and caregivers’ age were 58.6 ± 15.02 and 42 ± 15 years, respectively. The mean and standard deviation of the duration that the patients were having hemodialysis was 4.12 ± 3.74 years. About 52.5% of the patients (No: 129) were men. The caring ability of 53.1% of the patients (No: 131) was low, 35.9% (No: 88) was moderate, and only 11% of the patients (No: 27) had high levels of caring ability. Furthermore, 86.2% of the patients (No: 212), in addition to kidney disease, had other chronic diseases such as cardiovascular disease and diabetes. Also, 67.2% of the caregivers (No: 165) were women, 62.2% (No: 153) were married, 53.9% (No: 133) were housewives and 48.1% (No: 118) had under-diploma (high school) education (Table 1).

**Table 1:** Demographic characteristics of hemodialysis patients and their caregivers

Demographic characteristics of patients	
Variables	Total (*N* = 246)
Age (years), mean ± SD	58.6 ± 15
• Median (IQR)	60 (50–68)
Male gender, n (%)	129(52.5)
Care Ability	
• High	27(11)
• Medium	88(35.9)
• Low	131(53.1)
Having Other Chronic Diseases (yes: %86.2, n:212)	
• Cardiovascular	109(44.4)
• Diabetes	83 (33.7)
• Others	20 (8.1)
Demographic Characteristics Of caregivers of hemodialysis patients	
Variables	Total (*N* = 246)
Age (years), mean ± SD	42 ± 15
• Median (IQR)	40 (28–53)
Male gender, n (%)	81(32.8)
Monthly income (Dollar), n (%)^a^	
• < 100	148(60.2)
• 101–200	56(22.8)
• > 201	42(17)
Education, n (%)	
• Under Diploma	118(48.1)
• Diploma	85(34.6)
• College Education	43(17.3)
Job, n (%)	
• Housewife	133(53.9)
• Self-Employed	49(19.9)
• Employee	27(11.2)
• University Student	8(3.3)
• Unemployed	29(11.7)
Marriage, n (%)	
• Single	93(37.8)
• Married	153(62.2)

According to the recent surveys, monthly income of less than $ 540 in Iran means living below the poverty line, and the average salary in Iran is $ 220 per month

^a^Hemodialysis is free in Iran, and all related costs are paid by the government

The total score of care burden in caregivers was 64.8 out of 120. The score of care burden in the “time-dependence” domain was 17.6, in the “developmental” domain was 14.6, in the “physical” domain was 10.8, in the “social” domain was 12.08 and in the “emotional” domain was 9.7. The results showed that 42.7% of the caregivers (No: 105) had moderate level of care burden, 32.5% (No: 80) had a high level of care burden, 19.9% (No: 49) had low level of care burden, and 4.9% of them (No: 12) had a very high level of care burden.

There was a significant relationship between the age of caregivers and care burden (*P* < 0.001), so that, with an increase in the age of caregivers, the care burden also increased. Also, a significant relationship was found between the level of caregiver’s education and care burden (*P* < 0.001), so that, increased education level decreased care burden. With increasing capability of patient in self-care, the caring pressure of the caregiver decreased, which was statistically significant (*P* < 0.001). Also, there was a positive correlation between the patient’s incident of other chronic diseases and the care burden of caregivers (*P* < 0.001), so that, the care burden of caregiver who was caring for a patient with other chronic diseases was significantly higher than the caregivers whose patients did not have chronic diseases.

After analyzing the relationship between variables in univariate mode, in order to investigate the effect of each of the factors in the presence of other factors, variables with *p*-value of less than 0.2 entered into the multivariate covariance analysis (ANCOVA model) and finally, three variables including the caregiver age (*P* = 0.045), the patient capability in self-care (*P* < 0.001), and also the incidence of other chronic diseases (*P* = 0.022) remained in the ANCOVA model. Thus, these variables as factors influencing care burden were discussed in this study (Table 2). It should be noted that in multivariate analysis, the interaction between the remained last three variables was also evaluated and none of the interactions had a significant *P* value.

**Table 2:** Relationship between demographic characteristics and care burden in caregivers of hemodialysis

Quantitative variables	M ± SD	T	B	P*
Patient age	58.6 ± 15	−1.096	−.070	0.274
Caregiver age	42 ± 15	2.760	.178	0.006
Hemodialysis duration (years)	4.12 ± 3.74	−.046	−.003	0.963
Qualitative variables	M ± SD	df	F	P**
Patient gender				
Male	65.5 ± 18.07	1	0.459	0.499
Female	63.85 ± 19.89			
Caregiver gender				
Male	64.34 ± 18.60	1	0.109	0.741
Female	65.20 ± 19.19			
Monthly income (Dollar)				
< 100	67.32 ± 18.44	2	1.727	0.18
101–200	63.68 ± 18.76			
> 201	61.44 ± 20.94			
Caregiver education				
Under Diploma (less than 12th grade)	69.00 ± 18.72	2	4.152	0.007
Diploma (12th grade)	61.77 ± 18.94			
College Education	59.14 ± 17.57			
Caregiver job				
Housewife	65.56 ± 19.73	4	1.915	0.1
Self-Employed	59.85 ± 16.05			
Employee	65.85 ± 20.81			
University Student	58.12 ± 17.70			
Unemployed	71.07 ± 17.75			
Caregiver marriage status				
Single	63.26 ± 16.50	1	0.615	0.6
Married	65.77 ± 20.43			
Patient care ability				
High	48.04 ± 17.44	2	19.682	0.0001
Medium	58.35 ± 16.81			
Low	72.72 ± 16.74			
Having other chronic diseases				
Cardiovascular	61.60 ± 17.71	2	9.019	0.003
Diabetes	67.99 ± 18.81			
Others	73.50 ± 17.11			

*Based on linear regression

**Based on analysis of covariance

The mean and standard deviation of caregivers’ quality of life was 76.3 ± 13.7 out of 130. The score of quality of life in the “Physical domain” was 20.2, in the “Psychological domain” was 17.7, in the “Environment domain” was 23.3 and in the “Social Relationship domain” was 8.8.

Pearson correlation test showed a negative and significant correlation between the total scores of care burden and quality of life (r: -0.436; *P* < 0.001). Concerning the relationship between different domains of care burden and quality of life, the results indicated a negative and significant correlation between the environmental and the social domains of quality of life and all dimensions of care burden. The physical and psychological domains of quality of life had a negative and significant correlation with all dimensions of care burden, except for the time-dependence domain (Table 3).

**Table 3:** Correlation between dimensions of care burden and quality of life in caregivers of hemodialysis patients

Dimensions of care burden										
Dimensions of quality of life	Time-dependence		developmental		Physical		Social		Emotional	
	r^a^	95% CI	r^a^	95% CI	r^a^	95% CI	r^a^	95% CI	r^a^	95% CI
Physical	−0.044	(− 0.170 \ 0.82)	− 0.175	¶ (− 0.299 \ -0.050)	−0.242	¶ (− 0.364 \ -0.119)	−0.223	¶ (− 0.346 \ -0.100)	−0.261	¶ (− 0.383 \ -0.139)
Psychological	−0.096	(− 0.221 \ 0.030)	−0.327	¶ (− 0.446 \ -0.207)	−0.313	¶ (− 0.433 \ -0.193)	−0.300	¶ (− 0.420 \ -0.179)	−0.302	¶ (− 0.422 \ -0.182)
Environmental	−0.173	¶(− 0.297 \ -0.049)	−0.355	¶ (− 0.473 \ -0.237)	−0.382	¶ (− 0.498 \ -0.265)	−0.435	¶ (− 0.549 \ -0.322)	−0.355	¶ (− 0.473 \ -0.237)
Social	−0.176	¶(− 0.300 \ -0.052)	−0.379	¶(− 0.498 \ -0.262)	−0.346	¶ (− 0.465 \ -0.228)	−0.382	¶ (− 0.498 \ -0.265)	−0.257	¶ (− 0.379 \ -0.135)

¶ *P* < 0.05

^a^Pearson correlation coefficient

## Discussion

In our study, most caregivers experienced moderate to very high levels of care burden, which is consistent with many studies that have been investigating the care burden in caregivers of hemodialysis patients [3, 11, 20, 21]. Meanwhile, the results of several studies indicated that caregivers experienced relatively moderate level of care burden [22]. The level of care burden experienced by caregivers can be influenced by many factors such as governmental and non-governmental support of caregivers and dominant culture of society. In this regard and based on existing evidence [3, 10, 11, 21, 22], care burden in developing countries appears to be higher. In developing countries (such as Iran), compared with developed countries, the level of material and spiritual support provided for caregivers is not adequate [23]. Also, caring reactions, coping strategies, and attitude towards the care are influenced by culture. Accordingly, families are responsible for the care of patients due to the family structure in eastern societies like Iran [12]. In our opinion, the aforementioned factors may affect the level of care burden that caregivers experience in different countries and societies.

In our study, the caregivers’ quality of life was moderate. This finding is in line with a number of studies that have examined the quality of life in caregivers of hemodialysis patients. In one study, 52% of the caregivers of hemodialysis patients had moderate and low levels of quality of life [24]. Gill in a study compared the quality of life in caregivers of chronic renal failure patients in non-hemodialysis and hemodialysis patients. Quality of life in caregivers of patients who did not undergo dialysis was significantly higher than the other group, indicating a disorder in the quality of life of caregivers of hemodialysis patients [25]. The results of Shdaifat study, which examined the quality of life of caregivers of hemodialysis patients as well hemodialysis patients compared to the general population, showed that the average quality of life of patients and their caregivers was lower than the general public [26]. Although the relationship between care burden and quality of life of caregivers was not directly investigated in this study, the study referred to common complications of dialysis such as severe dietary restrictions, reduced social and recreational activities, medical complications, economic pressure, marital conflicts, sexual dysfunction, emotional stress, and anxiety as factors the affect the quality of life of patients and their caregivers. Also, the impact of hemodialysis on issues such as sleeping, eating, working, and planning for daily activities can be a challenge for patients and families, and together can lead to permanent changes in family roles and expectations, increased level of stress, and lower quality of life of individuals. Additionally, many of the problems associated with the care, such as changes in sleep patterns, social activity, and holiday plans and feelings of dependency on dialysis are among factors that affect the quality of life of caregivers [25, 26].

Abbasi [3] also reported a significant and inverse correlation between the total care burden and ability to perform personal activities, which is in line with the findings of this study. He also revealed a positive and significant correlation the existed between the total care burden and the duration of illness. In his study, caregivers with inappropriate economic status were significantly more prone to care burden [3].

Unlike the present study, Mashayekhi et al. [11] found a statistically significant relationship between the sex of the patients, their income and the level of care burden in their caregiver. Thus, the male caregivers and caregivers of low-income patients were experiencing higher levels of care burden. They also revealed no significant relationship between care burden and patient’s occupation, education, marital status, comorbidities and frequency of hemodialysis per week [11]. The differences in our findings and the results of Mashayekhi et al. study could be related to the difference in the research environment, amount of supporting facilities available to caregivers, and the relatively small sample size (51 caregivers) in the Mashayekhi et al. study. Bayoumi [10] reported a positive and significant correlation between the total care burden and patient’s age, but there was a significant but negative correlation between the total care burden and the caregiver’s age, patient’s level of education, and the caregiver’s level of education. The difference in our findings and the results of above study can be due to low sample size (50 caregivers) in the Bayoumi study, because low sample size reduces the level of transferability of study and also the chance to discover any correlation and association between variables.

In the current study, there was a significant and negative correlation between the total scores of care burden and quality of life, so that, the quality of life decreased with increasing care burden. Several studies suggested a negative correlation between care burden and quality of life in caregivers of hemodialysis patients [14, 26–28]. Mashayekhi reported a significant and inverse correlation between the quality of life and care burden in caregivers of hemodialysis patients [28]. Belasco found that caregivers of hemodialysis patients experience significant level of care burden that affects their quality of life [14]. Grant et al. concluded that care burden is also related to the quality of life of patients, so that, over time, reduced quality of life of the caregivers leads to decreased quality of life of patients [27]. We believe that, due to the chronic nature of hemodialysis and the high caring need of hemodialysis patients, their caregivers tolerate a high level of care burden, which inevitably leads to a decrease in the caregivers’ quality of life. In this regard, necessary support is required for caregivers of hemodialysis patients, and various governmental and non-governmental facilities and privileges should be provided to these people.

Our study encountered a number of limitations. The impossibility of determining the relationship between cause and effect due to the cross-secularity of the study was one of the limitations. The data collection method, which was based on self-reporting, might have also affected the outcome of the study. Despite the fact that, a desirable condition for measuring the level of care burden in caregivers of hemodialysis patients is to compare them with a control group at the community, the lack of control group in this study was one of our limitations and it is suggested to consider this issue in future studies. For future studies, it is suggested that, the relationship between care burden and quality of life in other countries should be studied and compared. Also, predictive factors of care burden and quality of life should be examined in caregivers of hemodialysis patients.

## Conclusions

In our study, caregivers of hemodialysis patients experienced a significant level of care burden that had a negative and significant correlation with their quality of life. Considering the effect that, care burden affects the quality of life of caregivers and their health as well as patient’s, comprehensive attention should be paid to the needs of caregivers and they should be provided by adequate social, economic, physical and psychological support.

## Abbreviation

KUMS: Kermanshah University of Medical Sciences
